# Completeness of colorectal cancer registration in the Danish hereditary non-polyposis colorectal cancer (HNPCC) register

**DOI:** 10.1007/s10689-025-00483-7

**Published:** 2025-06-28

**Authors:** Lars Joachim Lindberg, Inge Bernstein, Henrik Møller, Lone Sunde, Christina Therkildsen

**Affiliations:** 1 The Danish HNPCC Register, Gastro Unit, Copenhagen University Hospital – Amager and Hvidovre, Hvidovre, Denmark; 2 Department of Quality and Coherence, Hospital Management and Administration, Aalborg University Hospital, Aalborg, Denmark; 3 Department of Clinical Medicine, Aalborg University, Aalborg, Denmark; 4 Department of Clinical Medicine, Danish Center for Health Services Research, Aalborg University, Aalborg, Denmark; 5 The Danish Clinical Registries (RKKP), Aarhus N, Denmark; 6 Department of Clinical Genetics and Clinical Cancer Research Center, Aalborg University Hospital, Aalborg, Denmark; 7 Department of Clinical Research, Copenhagen University Hospital – Amager and Hvidovre, Hvidovre, Denmark

**Keywords:** Colorectal cancer, Registries, Data collection, Denmark, Lynch syndrome, Epidemiology

## Abstract

The Danish Hereditary Non-Polyposis Colorectal Cancer Register (HNPCC-R) has formed the basis for many epidemiologic studies supporting risk stratification and both national and international guidelines on surveillance. However, the HNPCC-R is based on voluntary reporting and the completeness of registration is unknown. Hence, we aimed to assess the validity and completeness of the colorectal cancer (CRC) registrations in the HNPCC-R. We combined the registrations in the HNPCC-R with the registrations in the national Danish Cancer Registry (DCR), which is validated and based on mandatory reporting, and matched the cases from each register using a 13-step algorithm based on unique, national Central Population Registration number, date of diagnosis, location, and morphology thus identifying 9160 verified CRCs in 49,799 individuals. The overall agreement between the registers was 85%, and the completeness of the HNPCC-R was 95%. The DCR had the highest number of registrations before 1975, and the HNPCC-R had the highest number of registrations after 1985—especially more synchronous and metachronous cases. In conclusion, data from the HNPCC-R on CRC are valid and should be preferred for studies on CRC in families with a heritable increased risk of colorectal cancer—especially in Lynch syndrome, which is known for multiple CRCs, though a combination of both registers would secure the most optimal dataset.

**Supplementary Information:**

The online version contains supplementary material available at 10.1007/s10689-025-00483-7.

## Introduction

The Danish Hereditary Non-Polyposis Colorectal Cancer Register (HNPCC-R) is a nationwide population-based register which has contributed substantially with data to multinational initiatives such as the Prospective Lynch Syndrome Database (PLSD) [1–3], the International Mismatch Repair Consortium (IMRC) [4], the international *PMS2* variant carrier study [5, 6] and an international study on Constitutional (bi-allelic) Mismatch Repair Deficiency (CMMRD) [7]. Research based on the data are used to direct international guidelines for clinical management of Lynch syndrome [8], which is a hereditary cancer-predisposition syndrome caused by a pathogenic variant in a mismatch repair (MMR) gene. HNPCC-R includes a unique collection of data on pedigrees, cancer diagnoses, pathogenic germline variants, and surveillance attendance and outcomes.

The HNPCC-R was established in 1991 and reached national coverage in 1993 where guidelines were published on how to identify the families and persons at risk and how to collect the required clinical data [9]. As the reporting was voluntary, there was a strong emphasis on the clinical impact of the HNPCC-R as this database could serve both as a clinical- and a research database with the aim to unify surveillance strategies in families dispersed across geographical regions as well as being the foundation for national and international research studies investigating the impact of surveillance [10]. The aims of the HNPCC-R still include both clinical management and research. However, the completeness of the colorectal cancer diagnoses has never been evaluated against a validated, national cancer database.

The national Danish Cancer Registry (DCR) is a well-established database with mandatory recordings since 1987. It has previously been validated against other Danish national databases and shown good agreement on cancer registration around 85–94% [11–13].

The aims of this study were to assess the agreement between colorectal cancer registrations in HNPCC-R and DCR, to estimate the completeness of colorectal cancer registrations in HNPCC-R, and to explore causes for disagreement.

## Materials and methods

### Registries

The Danish Central Population Register (CPR) was established in 1968 with the purpose to give all citizens in Denmark a unique personal identifier—a CPR number, which is used at all contacts with public authorities including health care and all private companies handling personal information such as social security, education, and income [14]. The CPR number allows for linking data in different registers on the same individual with 100% accuracy and without loss to follow-up if a citizen moves between different parts of Denmark.

The HNPCC-R was established with the purpose to decrease morbidity and mortality of cancer in individuals and families with a hereditary predisposition of colorectal cancer. In the early’90’ies, families with suspicion of a hereditary predisposition for colorectal cancer based on family history were included in the HNPCC-R. Since then, the genetic cause of Lynch syndrome has been discovered and the term “HNPCC” widely abandoned. However, the HNPCC-R kept its name and the families were classified according to their identified pathogenic germline variant or family cancer history using the three main groups: (1) Lynch syndrome due to a pathogenic or likely pathogenic variant in one of the four MMR genes *MLH1*, *MSH2*/*EPCAM*, *MSH6,* or *PMS2* regardless of family history; (2) Familial colorectal cancer with fulfilment or close to fulfilment of the Amsterdam I criteria defined by either a) three relatives with colorectal cancer (or two colorectal cancers and one adenoma with high grade neoplasia), where one relative is first degree to the two others, b) three relatives with colorectal cancer, where two is first degree, and one is second degree and diagnosed younger than 50 years, or c) two first-degree relatives with colorectal cancer, where at least one is diagnosed younger than 50 years; and (3) Moderate familial risk with either one family member diagnosed with colorectal cancer before age 50 or two first-degree relatives diagnosed ≥ 50 years [15].

Reporting to HNPCC-R is voluntary but has gained national outreach—especially accelerated by the co-financed EU-project InfoBioMed in 2007 [16]), where registrations are done electronically. Clinical data are collected from surgeons, pathologists, clinical geneticists, colonoscopists, and genetic laboratories. Variables recorded include data on an individual level (such as CPR number, sex, genetic tests, surveillance procedures, cancers, and polyps including histopathological analyses) and data on a family level (such as risk classification, recommended surveillance programme, and pedigree).

Relevant relatives are checked manually in other relevant registers for cancers and surveillance procedures. Cancer diagnoses are verified at the HNPCC-R and type of documentation are coded as pathology report, medical record, death certificate, or “family report” if information given by a member of the family cannot be verified in any of the former three resources. If the family reports a condition that they think is colon cancer, but the condition can be verified to be a benign condition e.g. as diverticulitis coli, it is deleted.

Data are collected systematically at entry into HNPCC-R (retrospectively) and is continuously up-dated (prospectively), e.g., when a family member consults a department of clinical genetics, or when new cancers are reported to the HNPCC-R. Registrations of colorectal cancer include date of diagnosis, ICD9 code, location, morphology, surgical procedure, and type of documentation. Date of diagnosis is defined as date of cancer surgery, date of biopsy for unresectable tumors, or best estimated date if diagnoses are based on death certificates.

The DCR was founded in 1943 as a national cancer register with the aim to collect data enabling reliable incidence statistics across time, area, occupation, and therapeutic approach [17, 18]. The register includes data on primary cancers through medical records and pathological reports secured by computerized and visual quality control routines [18]. The reporting from hospital departments was initially voluntary, but was made compulsory at March 1987 [19]. In 2004, the data recording was automated with only 10% of diagnoses collected through manual registration and contact to relevant clinical departments. The register has a high data completeness gained through annual linkage with other national registers such as the National Patient Register, the Danish Pathology Register, and with death certificates [20]. DCR collects data on incident carcinomas, sarcomas, leukemias, and lymphomas as one diagnosis per organ with exceptions made for multiple cancers of the skin and paired organs with similar morphological characteristics [18, 21]. “One diagnosis per organ” are defined by ICD codes allowing for registrations of synchronous and metachronous colorectal cancer in different colonic segments as each segment of the colorectum has a unique ICD code. Variables recorded in DCR include CPR number, sex, date of diagnosis, ICD7/ICD10 code, location, and morphology. Date of diagnosis is defined as the date of first admission during which the cancer was diagnosed [20].

### The study population

The study period ranged from January 1943 to December 2014. The study population included members of families registered in HNPCC-R at or before the 20th of February 2018. For families with Lynch syndrome, we included carriers of a (likely) pathogenic variant (n = 1798), non-carriers (n = 2360), and untested relatives with a 50% risk of carrying the variant (n = 3925). For familial colorectal cancer and moderate familial risk, we included individuals at risk defined as colorectal cancer patients and their first-degree relatives (n = 26,800 and n = 14,916, respectively). Only individuals with a valid CPR number were eligible for the study.

### Matching of colorectal cancer registrations

Colorectal cancer registrations were retrieved from both the HNPCC-R and the DCR and matched by CPR numbers.

ICD7 and ICD10 diagnoses from the DCR were converted to ICD9 diagnoses to match the diagnoses in HNPCC-R before data were merged in SAS [22] by CPR numbers, creating all possible combinations of registrations in the two registers on the same individual. To judge which combinations correctly matched the same colorectal cancer, the registrations in each combination were compared using a 13-step algorithm to match the diagnoses as correctly as possible in patients with multiple diagnoses (Supplementary Table 1). In brief, for each patient colorectal cancer registration(s) from both registers were matched by date of diagnosis, localization, and morphology with stepwise less agreement, ranging from maximum of 12 months difference between the date of diagnosis in identical segments of the colon, to more than 12 months difference and in adjacent segments. For simplicity, all the cases that could be matched by one of the 13 steps in the algorithm were pooled and referred to as matched cases. Detailed matching criteria are presented in Supplementary Table 1.

Registrations in DCR were considered as matched if they could be matched with a registration in HNPCC-R up to one year after the end of the study period to account for dates in DCR systematically being registered earlier in time than dates in HNPCC-R (because dates in DCR are date of first admission and dates in HNPCC-R are date of treatment).

Registrations of colorectal cancer diagnoses in one register without a match in the other register were checked for possible matches with registrations of other neoplasia instead, and in case of synchronous registrations of different neoplasia in the two registers the original documentation in HNPCC-R was reevaluated to reveal possible errors in registration of diagnoses, localization, or histology in one of the registers. Errors in the HNPCC-R were corrected in the process. Registrations of colorectal cancers in HNPCC-R without a matching registration in DCR were also manually checked by reevaluating the original documentation. We could not retrieve documentation for the cases only found in DCR.

### Statistics

Number of unique colorectal cancer registrations was defined by the number of cases registered in both HNPCC-R and DCR plus the number of cases only registered in one of the registers.

Agreement between HNPCC-R and DCR was defined by the number of colorectal cancer registrations in both registers divided by the number of unique registrations.

Completeness of a register was defined by the number of colorectal cancer registrations in the register divided by the number of unique registrations.

*p*-values were estimated in SAS with the Chi-square test—or Fisher’s exact test if group sizes were smaller than 5. Adjusted *p*-values were estimated with a stepwise logistic regression analysis in SAS with the proc logistic function.

## Results

**Fig. 1:**
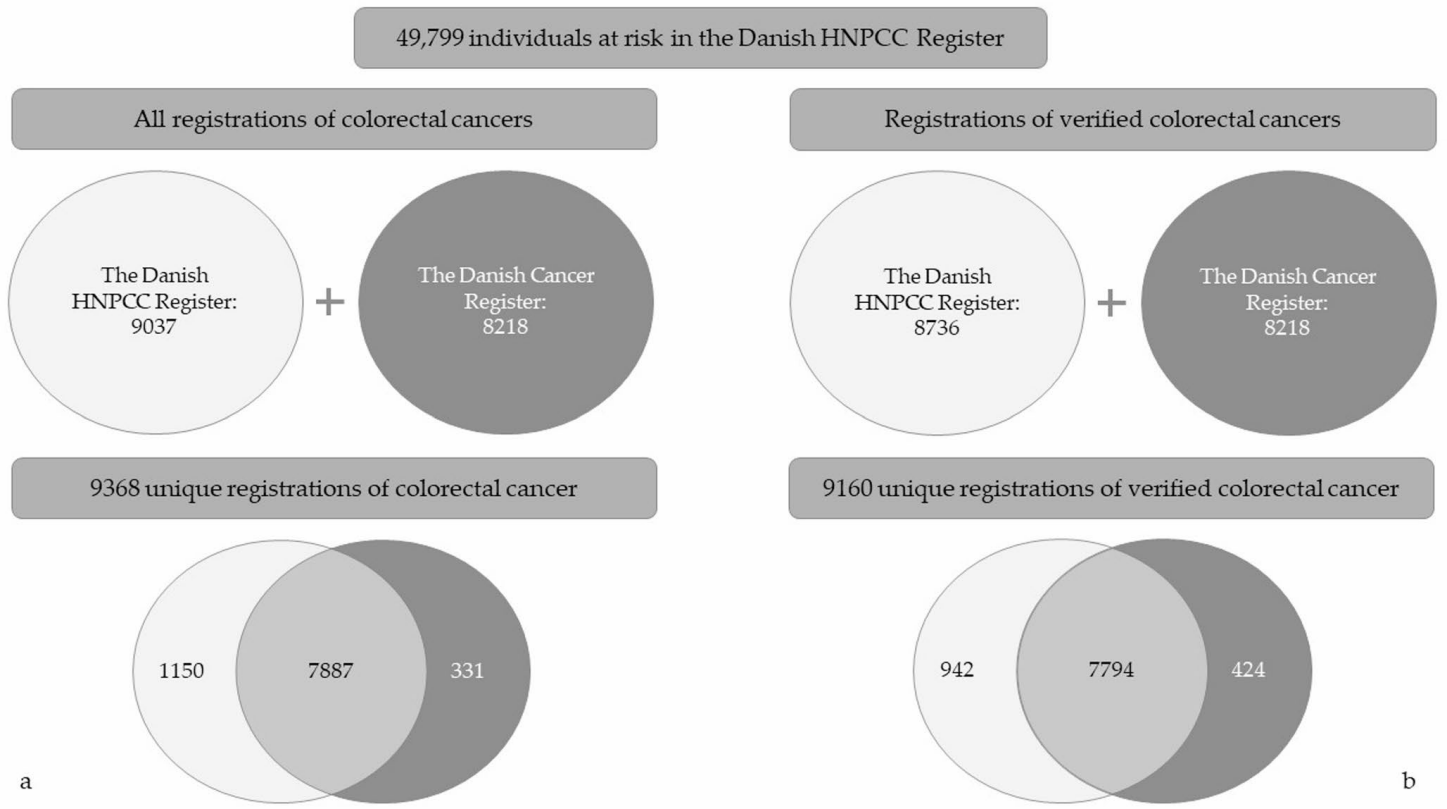
**a** and **b** Cohort and registrations of colorectal cancer in the Danish HNPCC Register and/or the Danish Cancer Registry

From the HNPCC-R, 49,799 at-risk individuals were identified, and colorectal cancer diagnoses were retrieved from each register. Within the study population, 9037 colorectal cancer registrations were found in the HNPCC-R and 8218 in the DCR (Fig. 1a).

### Agreement between the two registers

Matching the colorectal cancer registrations in the two registers identified 9368 unique colorectal cancer registrations of which 7887 were found in both registers, resulting in an agreement of 84.2% (Fig. 1a). The highest agreement between the registers was observed for colorectal cancer registrations verified by death certificates (91.7%) and pathology reports (89.8%) followed by medical records (78.6%) (Table 1). Agreement on cancer diagnoses only based on family reports was very low (30.9%), and these cases were therefor excluded from further analyses, leaving 9160 verified colorectal cancers in 8248 patients (Fig. 1b).

**Table 1:** Agreement on registration of colorectal cancer between the Danish HNPCC Register (HNPCC-R) and the Danish Cancer Registry (DCR) by type of documentation in HNPCC-R

	Agreement		*p*-value
	[%]	[n]	
Total	87.3	(7887)	< 0.0001
Type of documentation			
Pathology report	89.8	(6790)	
Medical record	78.6	(437)	
Death certificate	91.7	(567)	
Family report	30.9	(93)	

Based on registrations of verified colorectal cancer, the overall agreement between HNPCC-R and DCR was 85.1% with 942 registrations only found in HNPCC-R and 424 only found in DCR (Table 2). Agreement according to the 13-step algorithm are shown in Supplementary Table 1.

**Table 2:** Agreement on registration of colorectal cancer (CRC) between the Danish HNPCC Register (HNPCC-R) and the Danish Cancer Registry (DCR)

	Agreement		*p*-value	
	[%]	[n]	Raw	Adjusted
Total	85.1	(7794)	< 0.0001	0.66
Risk classification				
Lynch syndrome	74.8	(975)		
FCC	86.3	(4538)		
MFR	87.9	(2281)		
Period			< 0.0001	< 0.0001
1943–1954	75.0	(27)		
1955–1964	58.3	(63)		
1965–1974	78.0	(500)		
1975–1984	85.6	(1002)		
1985–1994	87.0	(1511)		
1995–2004	86.5	(2167)		
2005–2014	85.2	(2524)		
Number of CRCs			< 0.0001	< 0.0001
First	89.7	(7407)		
Syn-/metachronous	42.7	(387)		
Timing of CRC			< 0.0001	< 0.0001
Retrospective	86.3	(7361)		
Prospective	68.7	(433)		

*FCC* Familial colorectal cancer, *MFR* Moderate familial risk of CRC

**Fig. 2:**
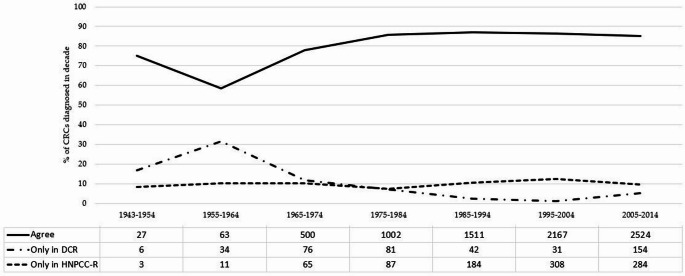
Agreement on registration of colorectal cancer (CRC) between the Danish HNPCC Register (HNPCC-R) and the Danish Cancer Registry (DCR) over time

The HNPCC-R is a dynamic register with new families being added continuously and thus continuously growing in number of individuals at risk—and in number of colorectal cancer cases over time (Fig. 2).

### Completeness of the HNPCC-R

The overall completeness of verified colorectal cancer registrations in HNPCC-R was 95.4% (Table 3) which was significantly higher than in the DCR (*p* < 0.001). The completeness in the HNPCC-R was similar across risk classifications of the families with 93.3% completeness in Lynch syndrome, 95.8% in familial colorectal cancer, and 95.5% in moderate familial risk of colorectal cancer (Table 3).

**Table 3:** Completeness of registrations of colorectal cancer in the Danish HNPCC Register (HNPCC-R) and the Danish Cancer Registry (DCR)

	Completeness (95% CI)		*p*-value	Only in HNPCC-R		Only in DCR	
	HNPCC-R	DCR		[%]	[n]	[%]	[n]
Total	**95.4** (94.9–95.8)	**89.7** (89.1–90.3)	< 0.001	10.3	(942)	4.6	(424)
Risk classification			0.016				
Lynch syndrome	**93.3** (91.8–94.6)	**81.4** (79.2–83.5)		18.6	(242)	6.7	(87)
FCC	**95.8** (95.2–96.3)	**90.5** (89.7–91.2)		9.5	(501)	4.2	(221)
MFR	**95.5** (94.7–96.3)	**92.3** (91.2–93.3)		7.7	(199)	4.5	(116)
Period			< 0.001				
1943–1954	**83.3** (68.1–92.1)	**91.7** (78.2–97.1)		8.3	(3)	16.7	(6)
1955–1964	**68.5** (59.3–76.5)	**89.8** (82.7–94.2)		10.2	(11)	31.5	(34)
1965–1974	**88.1** (85.4–90.4)	**89.9** (87.3–92.0)		10.1	(65)	11.9	(76)
1975–1984	**93.1** (91.5–94.4)	**92.6** (90.9–93.9)		7.4	(87)	6.9	(81)
1985–1994	**97.6** (96.7–98.2)	**89.4** (87.9–90.8)		10.6	(184)	2.4	(42)
1995–2004	**98.8** (98.2–99.1)	**87.7** (86.4–88.9)		12.3	(308)	1.2	(31)
2005–2014	**94.8** (93.9–95.5)	**90.4** (89.3–91.4)		9.6	(284)	5.2	(154)
Number of CRCs			< 0.001				
First	**95.7** (95.2–96.1)	**94.1** (93.6–94.6)		5.9	(487)	4.3	(359)
Syn-/metachronous	**92.8** (91.0–94.3)	**49.8** (46.6–53.1)		50.2	(455)	7.2	(65)
Registration in HNPCC-R			< 0.001				
Retrospective	**96.5** (96.1–96.9)	**89.8** (89.2–90.4)		10.2	(869)	3.5	(300)
Prospective	**80.3** (77.0–83.2)	**88.4** (85.7–90.7)		11.6	(73)	19.7	(124)

*CRC* Colorectal cancer, *DCR* The Danish Cancer register, *FCC* Familial Colorectal Cancer, *HNPCC-R* The Danish HNPCC register, *MFR* Moderate Familial Risk of CRC

Bold has been chosen to highlight primary results from confidence intervals to improve readability of the table

### Causes for disagreement

Time influenced the agreement between registrations in HNPCC-R and DCR (*p* < 0.001) (Table 2 and Fig. 2) with poor agreement in the early decades of the study period and a stable agreement above 85% after 1975. Likewise, completeness changed over time with DCR having the highest number of registrations before 1975 regarding colorectal cancer registrations and HNPCC-R having the highest number of registrations since 1985 (Table 3 and Fig. 2).

Agreement between the two registers were affected by the number of colorectal cancer registrations per patient with 89.7% agreement on registrations of the first colorectal cancer, and only 42.7% agreement on registrations of synchronous and metachronous colorectal cancers (*p* < 0.001) (Table 2). The raw agreement was lowest in Lynch syndrome, but the difference was not significant after adjusting for number of colorectal cancers per patient, retro- or prospective registration in the HNPCC-R and period of diagnosis (adjusted *p*-value 0.66) (Table 2). Likewise, completeness of registrations of the first colorectal cancer in a patient was 95.7% in HNPCC-R and 94.1% in DCR, but synchronous and metachronous diagnoses were registered significantly more frequently in HNPCC-R (92.8%) than in the DCR (49.8%, *p* < 0.001) with 455 synchronous or metachronous colorectal cancers only registered in HNPCC-R (Table 3).

Agreement between the two registers were also affected by date of diagnosis being before or after inclusion of the patient in the HNPCC-R with lower agreement between the registers for prospective colorectal cancer registrations reflecting 124 cases only recorded in DCR (Table 3). Likewise, completeness of colorectal cancer registrations retrospectively collected by the HNPCC-R was higher than for the prospectively collected diagnoses (96.5% versus 80.3%, *p* < 0.001) (Table 3).

Disagreement between the two registers was explained in 199 cases by an incorrect or incomplete registration of CRC in one of the registers: incorrect registration of organ of origin (n = 36) e.g. uterine cancer registered as colorectal cancer or vice versa; incorrect registration of histology (n = 55) e.g. colorectal carcinoids registered as adenocarcinomas or vice versa; and incomplete registrations (n = 108) e.g. colorectal cancers recorded as unspecified cancer or metastases recorded without primary cancer (Supplementary Table 2). Only 25 cases were incorrectly recorded as colorectal cancers, when in fact being extra-colorectal cancers. Of these, 20 (0.2%) cases were found in the HNPCC-R among the 8736 colorectal cancer registrations.

## Discussion

In this study comparing two national registers, we found that the HNPCC-R had a high agreement with the DCR regarding registrations of colorectal cancer (85.1%), despite HNPCC-R being based on voluntary reporting and DCR being based on mandatory reporting. In addition, we found that the HNPCC-R had a high completeness of colorectal cancer registrations (95.4%) with 942 cases only registered in the HNPCC-R compared to the DCR, and that the DCR only had 424 cases unknown to the HNPCC-R. Previous studies comparing the DCR with the Danish Colorectal Cancer Group Database, the Danish Lung Cancer Register, and the Danish Renal Cancer Database have shown similar agreements between the registers (85–87%) and similar or lower completeness of the cancer-specific registers (88–96%), despite all databases used the same primary data sources and did not rely on voluntary reporting [11–13]. The high completeness observed in the HNPCC-R may be attributable to the fact that the register was initiated in 1991 by idealistic specialists at the department of gastroenterological surgery, who were performing surgery on the patients, identifying their high risk of colorectal cancer, surveilling them afterwards, and following them through genetic counselling in the early 1990’ies. Since then, focus has been on both being clinically and academically relevant by i.e. identifying relatives at risk, coordinating surveillance across the country, participating in revisions of clinical guidelines, sharing data with contributors for research purposes, and host an annual symposium for all with interest in HNPCC, thus encouraging contributors to keep reporting data to the HNPCC-R.

Missing data may be explained by different foci of the registers, different establishment dates of the registers, and different procedures for data acquisition. From the initiation of the HNPCC-R, focus included synchronous and metachronous colorectal cancer, and national and international research studies based on data from the HNPCC-R showed that HNPCC patients had a high risk of both multiple and interval cancers [10, 23, 24] which further motivated recordings of synchronous and metachronous colorectal cancer. In contrast, the DCR was established to evaluate the incidence of cancer [17, 19] and though it is possible to record multiple cancers in this register, DCR follows international guidelines to only record synchronous and metachronous cancers that are sufficiently different from earlier recordings, defined by difference in segmental location in the colorectum or a completely different morphology. Hence, in cases with e.g. two adenocarcinomas in the cecum, only one cancer will be recorded in the DCR. These guidelines secure that two pathology reports on the same cancer are not recorded as two cancers, and that a recurrent cancer is not recorded as a metachronous cancer, resulting in an under-registration of multiple cancers [21]. The different foci of the two registers have likely contributed to the low agreement regarding syn- and metachronous CRCs, which in turn explains the difference between the raw and adjusted *p*-values in Table 2 as Lynch syndrome families have more synchronous and metachronous colorectal cancer than other families.

Different establishment dates and procedures for data acquisition between the registers could also explain some of the differences in registered colorectal cancer cases. The DCR was established approximately 50 years before the HNPCC-R and has prospectively recorded cancers reported by medical doctors and later by automated retrieval of cancer data from other national registers [18, 20]. In contrast, data in the HNPCC-R have been entered and verified retro- and prospectively by manually retrieving pathology reports, medical records, and death certificates from sources with different initiation dates and varying registration protocols through time. In Denmark, death certificates are never deleted but pathology reports could be deleted after 10 years until establishment of the Danish Pathology Register in 1997 [25] and medical records can still be deleted after 10 years, which makes it increasingly difficult for the HNPCC-R to retrospectively verify cancers with increasing age of the cases—and thus likely explains the lower completeness in HNPCC-R compared to DCR before 1975. Cases of cancer reported by a member of the family, which cannot be verified in pathology reports, medical records, or death certificates are in the HNPCC-R recorded with the documentation type “family report” and may be used clinically to identify individuals at risk but have usually not been included for research purposes. In this study, only 31% of cases with documentation type “family report” in the HNPCC-R could be verified by a match in the DCR, supporting the practice in the HNPCC-R with not including unverified records for research. Exclusion of the 93 unverified records in the HNPCC-R that could be verified in the DCR in this study corresponds to an under-registration of 1.1%, which we find acceptable and clinically irrelevant.

Finally, missing data could also be explained by the lack of automated updates of cancers in the HNPCC-R during the prospective part of the data collection. This can be seen in a completeness of colorectal cancer registrations of 97% when only retrospectively collected cases are considered compared to 80% focusing only on prospectively collected data (Table 3). As prospectively registered data in the HNPCC-R are added continuously when families are clinically revisited, data completeness will increase with time since diagnosis.

As a result of the difficulties in finding and verifying cancers, one study limitation is the risk of colorectal cancer cases missed in both registers, resulting in overestimation of the completeness. As these cancers may not even be found in the Danish Pathology Register, linkage to this register was not sought. On the other hand, we compared the HNPCC-R with the previously validated DCR with mandatory registration and a very high completeness [11–13] and investigated disagreement between the registers via retrieval of the original pathology reports, death certificates and medical records on the colorectal cancers. Another limitation is, that the 13-step algorithm for matching of colorectal cancer registrations from the two registers has not been validated, but we assume over-estimation of agreement to be very low, as the CPR numbers secure that matching is exclusively performed on data regarding the same individual.

Lessons learnt from this study include that completeness of cancer registration is facilitated by 1) a unique personal identifier to link data from different registers and prevent dropouts, 2) mandatory reporting of cancers, 3) focus on syn- and metachronous cases, 4) procedures to secure prospective updates of new cases in already registered individuals.

## Conclusion

There was a good agreement between the registers, although with time differences where colorectal cancer registrations were more complete in the DCR from 1943 to 1975 and more complete in the HNPCC-R since 1985—especially regarding synchronous and metachronous cases which are of high relevance in hereditary cancer disposition.

Data from the HNPCC-R on colorectal cancer are valid and should be preferred for studies on colorectal cancer in families with a heritable increased risk of colorectal cancer—especially in Lynch syndrome, which is known for multiple colorectal cancers, though a combination of both registers would secure the most optimal dataset.

## Electronic supplementary material

Below is the link to the electronic supplementary material.

Supplementary Material 1

Supplementary Material 2

## Data Availability

The Danish HNPCC register hosts a database including clinical, pathological and genetic data available to both Danish and international researchers through collaboration. A multidisciplinary scientific board ensures transparency in the process of making data available for research. The data that support the findings of this study are available from the authors but restrictions apply to the availability of these data, which were used under license from the national Danish Cancer Registry for the current study, and so are not publicly available. Data are, however, available from the authors upon reasonable request and with permission from the national Danish Cancer Registry.

## Declarations

### Dual publication

The results/data/figures in this manuscript have not been published elsewhere, nor are they under consideration (from you or one of your Contributing Authors) by another publisher.

### Third party material

All of the material is owned by the authors and/or no permissions are required.
