# Supplementary figures and images for: Completeness of colorectal cancer registration in the Danish hereditary non-polyposis colorectal cancer (HNPCC) register
Source: Fam Cancer. 2025 Jun 28;24(3):57. doi: 10.1007/s10689-025-00483-7 (PMC12206165; doi:10.1007/s10689-025-00483-7)

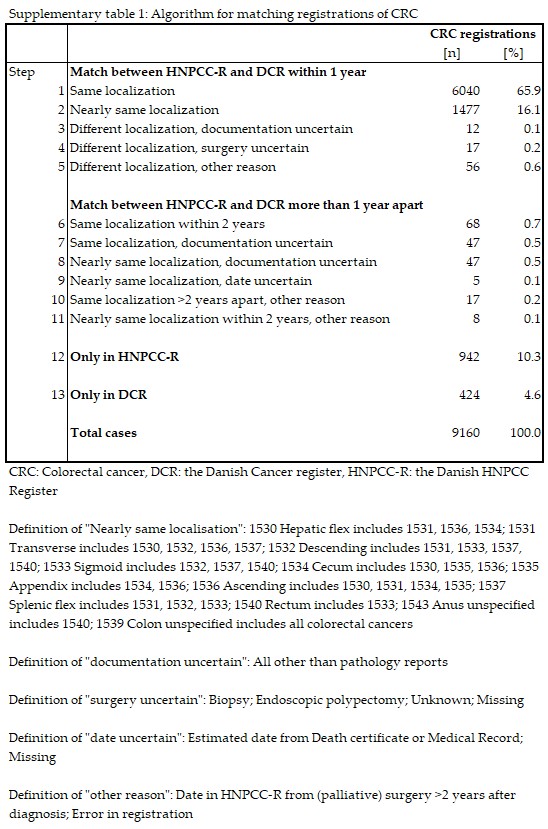

Supplement: Supplementary file 1 — Supplementary Material 1 [file 10689_2025_483_MOESM1_ESM.jpg]

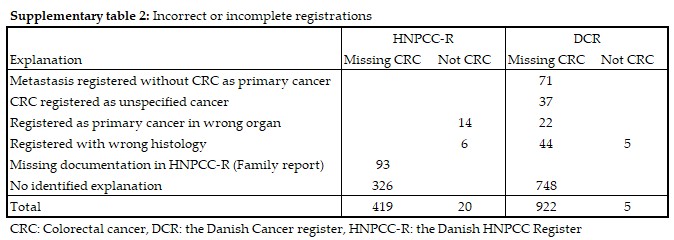

Supplement: Supplementary file 2 — Supplementary Material 2 [file 10689_2025_483_MOESM2_ESM.jpg]
